# The role of emptying services in provision of safely managed sanitation: A classification and quantification of the needs of LMICs

**DOI:** 10.1016/j.jenvman.2021.112612

**Published:** 2021-07-15

**Authors:** Nicola Greene, Sarah Hennessy, Tate W. Rogers, Jocelyn Tsai, Francis L. de los Reyes III

**Affiliations:** a Triangle Environmental Health Initiative, LLC, Durham, NC, USA; b Department of Civil, Construction, and Environmental Engineering, North Carolina State University, Raleigh, NC, USA

**Keywords:** Sanitation, Onsite, Pit latrine, Septic tank, Africa, Asia

## Abstract

Classifications for onsite sanitation in terms of facility type (septic tanks, pit latrines) exist, but connecting these facilities to the wider sanitation value chain via improved containment, emptying, and collection has not been well explored. Using existing Joint Monitoring Programme facility classifications and secondary data on piped water access, a Service Typology was developed to classify and quantify the primary emptying service needs of household level onsite sanitation facilities. Facilities in six Sustainable Development Goal (SDG) regions were classified as Emptiable (faecal sludge can be removed either via Mechanized or Non-Mechanized means) or Unemptiable. Of the 722 million household level sanitation facilities assessed in these regions, 32% were found to be emptiable via Mechanized means, 50% via Non-Mechanized means and 18% were found to be Unemptiable pits. The volume (by number of facilities) and density (as a proportion of the full population) of each service type were estimated by SDG region and by country. Results from this study provide background data on the role of emptying sanitation facilities in achieving SDG6, and can be incorporated into investment priorities, policy framing, technology development, infrastructure development, and targeted behaviour change strategies.

## Highlights

• First study to quantify the service needs for 772 M onsite sanitation facilities.
• 32% of facilities are emptied via Mechanized means using pumps or vacuum trucks.
• 50% of facilities are manually emptied via Non-Mechanized means.
• 18% of facilities are unimproved and Unemptiable due to their poor quality.
• Service innovation for dry, improved onsite facilities identified as priority need.

## Graphical abstract

**Figure:**
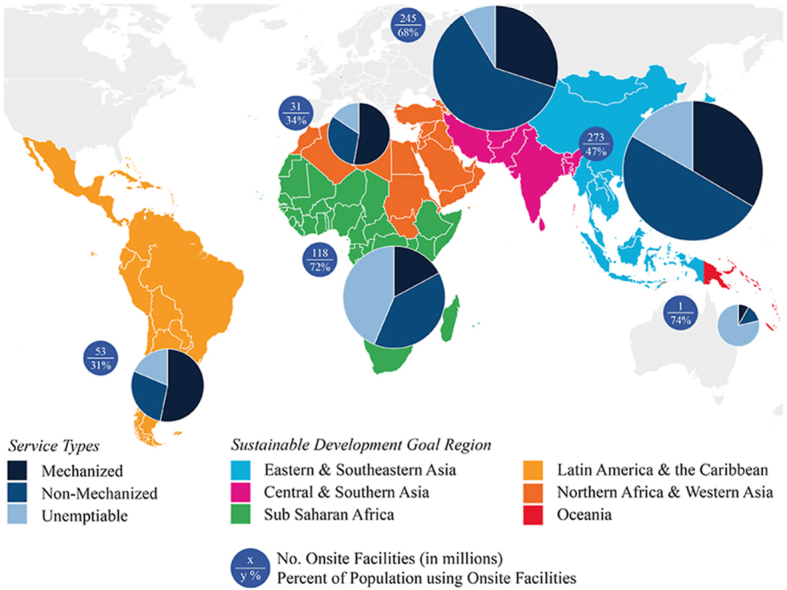

## 1 Introduction

By necessity or by choice, many countries depend on ‘onsite’ sanitation facilities: systems *“in which excreta and wastewater are collected, stored and/or treated on the plot where they are generated*” (Jenkins et al., 2015; Tilley et al., 2014). In low-and middle income countries (LMICs), the high costs associated with sewer systems and centralized wastewater treatment, the unavailability of water for flushing and conveying waste, the lack of space for sewer lines in dense cities and settlements, and other infrastructure capacity challenges, are contributing to increasing clarity that sewers may not be a feasible or favourable sanitation solution for LMICs (Bassan and Strande, 2013; Capone et al., 2020; Mikhael et al., 2018). Citywide Inclusive Sanitation (CWIS) embraces these findings by encouraging a shift in focus for urban sanitation planning, by promoting a package of solutions tailored to local contexts, which often do not include sewers (Gambrill et al., 2020).

While onsite sanitation containment technologies have been classified and described (Tilley et al., 2014), one of the key links in the sanitation chain-faecal sludge containment, emptying, and collection – is often neglected in regional or global analyses (Strande, 2014; Tremolet, 2013). Onsite sanitation systems accumulate waste in situ and must either have faecal waste removed and transported to a treatment facility, or disposed at or near the site (typically via abandoning the pit or removing the contents and burying them) (Gold et al., 2014; Thye et al., 2011). Safely managed sanitation requires that “*excreta (are) safely disposed either* in situ *or transported and treated offsite*” (*Sanitation*, *JMP*, 2017), with the ability to empty the containment facility as a crucial first step. This emptying step depends on a variety of factors, including facility type, access to the site, level of trash (undesirable solid waste) dumped inside pits and tanks, sludge thickness and viscosity, and quality of the onsite system, among others.

There are two main technology sub-categories of onsite systems which dictate the contents of the containment facilities: ‘wet’ technologies which require water for flushing (Mills et al., 2014); and ‘dry’ technologies which do not require any water for flushing (Tilley et al., 2014). This categorization plays a key role in determining whether a mechanized system for pit emptying (e.g., using pumps, or something that can be considered ‘machine-like’) is feasible.

A traditional mechanized approach is the use of vacuum trucks that are equipped with a vacuum pump designed to remove liquids, sludges, and slurries from below ground into the tank of the truck for transport to another location. However, there are logistical and technical challenges that limit the universal use of vacuum trucks. These challenges include: (i) dry contents – pit latrines may be unlined or have low levels of water added, resulting in contents that lie outside the operational range of existing pumps; (ii) trash – in the absence of solid waste disposal services, the onsite sanitation facility can be used as a de-facto dustbin (Still, 2002), and trash can clog vacuum lines; (iii) access – with growing use of onsite sanitation facilities in densely populated urban areas, vacuum trucks cannot get close to the pits due to lack of space, road access or elevation challenges (Still et al., 2013; Strande, 2014; Sisco et al., 2017); and (iv) low quality– unlined containment facilities that risk collapse if contents are removed rapidly by vacuum trucks; millions of people have such low quality pit latrines (Chunga et al., 2016; Chiposa et al., 2017).

Because of these challenges, many onsite sanitation facilities are either abandoned (considered unemptiable) or emptied in a non-mechanized or manual manner. While the process of abandoning an onsite facility can be carried out safely in certain circumstances, it is a disincentive for the household to invest in a facility knowing that it has a limited lifespan. On the contrary, manual or non-mechanized emptying can rarely be considered safe. Manual removal of a faecal sludge containment facility is labour intensive, time consuming, typically performed at significant risk to the service provider, and in many countries illegal because of the undignified and hazardous work conditions (Capone et al., 2020). The practice is sometimes known as ‘postponed open defecation’ due to the fact that waste is often indiscriminately disposed of in a hazardous manner (Verhagen and Carrasco, 2013). Pockets of knowledge exist on this practice, but its prevalence on a global scale is yet to be quantified.

This study classifies the faecal sludge emptying market into service types and uses the typology to quantify the product and service needs for enabling safe faecal sludge removal from onsite sanitation facilities. This study prioritises LMICs and the analysis is concentrated in six Sustainable Development Goal (SDG) regions: Central and Southern Asia, East and Southeastern Asia, Latin America and the Caribbean, North Africa and Western Asia, Oceania and Sub Saharan Africa. Using available secondary data on facility types and water access, emptying markets are described both in terms of scale and the density of need. The purpose of this work is to categorise and quantify emptying needs to better understand the priorities in achieving global access to safely managed sanitation. The data and analysis from this work can be applied to create enabling environments for safely managed onsite sanitation by identifying needs in parallel infrastructure, policy, regulation, technology, behaviour change programs, funding, and the development of public and private partnerships.

## 2 Materials and methods

Data from the WHO/UNICEF Joint Monitoring Programme for Water Supply, Sanitation and Hygiene (*Home | JMP*, n.d.), collected in 2017 and published in 2019, were used to estimate the physical number of household onsite sanitation facilities in the regions of interest, and allocate the onsite sanitation facilities to service types.

### 2.1 Estimating the number of onsite sanitation facilities

#### 2.1.1 Secondary data sources - sanitation access

JMP data describing the facility types (sewer, septic tank, improved latrine and other) and service levels (open defecation, unimproved, basic service, at least basic, limited and safely managed) (Fig. 1 in the Supplementary Information) in urban and rural populations at the household level were accessed at https://washdata.org/data/household. Because JMP data do not distinguish between urban and rural in their ‘total’ calculations, we used the sum of the JMP urban and rural population data as the ‘total’ number approximations, to allow analysis with an urban/rural lens.

**Fig. 1:**
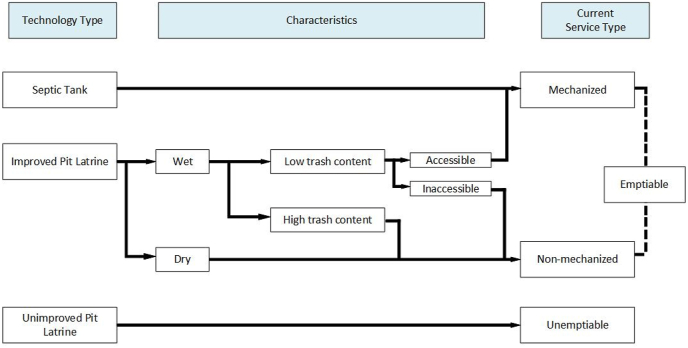
The conversion of the JMP Technology Types of onsite sanitation facilities into Emptying Service Types. The ease of sludge removal from each technology type is characterized by the facilities i) water content - ‘wet’ and ‘dry’ sludge, ii) trash content, and iii) accessibility. The Service Types designate whether a facility is emptiable so that the sanitation facility may be re-used and sludge safely managed.

While data available on improved sanitation facilities are disaggregated as septic tanks and improved pit latrines, data on unimproved facility types are not disaggregated, and facilities in this category are all considered as one facility ‘type.’ Populations practicing open defecation welure assumed to lack a sanitation facility, and therefore not included in this analysis. As such, the onsite sanitation analysis used the following facility levels: septic tanks, improved pit latrines, and unimproved facilities.

Data in the JMP are provided in terms of population (e.g., number of people using septic tanks). To calculate the number of facilities in a country, the population data was divided by the average household size of the country. Data for each country's 2017 population and average household size were obtained from the United Nations (UN) Department of Economic and Social Affairs (Household: Size and Composition 2018 *- Countries*, n.d.). Where average household sizes were unavailable for a specific country, the average household size for the SDG region was used.

An incongruity in this method arose in considering *shared* sanitation facilities. Shared sanitation facilities are considered ‘Limited’. To estimate the number of shared facilities, country population proportions of unshared to shared (basic/at least basic + safely managed compared to limited) improved sanitation facilities were calculated and distributed across the three facility types (sewers, septic tanks, improved latrines). To convert the resulting population figures to the number of onsite facilities, an assumed sharing rate of three households was selected (Gunther et al., 2012; Norman and Schelbert, 2018) and the number of improved shared facilities was calculated using Equation (1):

(1) $$No.\;of\;Shared\;Facilities=\frac{Population\;using\;Shared\;Facilities}{3\;x\;Average\;Household\;Size}$$

The number of shared, *unimproved* facilities was calculated by taking the same unshared to shared proportion from improved facilities and applying the ratio to the unimproved category to calculate the population in this segment. Equation (1) was also used to calculate the number of unimproved shared facilities. This potentially led to an underestimation of the number of shared facilities, because lower income populations using unimproved facilities could be more prone to sharing. What results is a potential fractional overestimation in the number of unimproved facilities, brought about by an underestimation of the proportion of facilities that may be shared (see Sensitivity Analysis, Table 2, in Supplementary Information).

### 2.2 Allocation of service types

The ease of removing faecal sludge from an onsite facility is a function of three key determinants: (i) trash content in the pit; (ii) accessibility of the facility; and (iii) whether the facility is wet or dry. A ‘wet’ pit is defined as one where the average total sludge solids content of the pit is low enough for the contents to be pumped using conventional mechanical means such as vacuum trucks and off the shelf pumps. In contrast, ‘dry’ pits cannot be pumped using mechanical means. A rheological study of faecal sludge has shown that below 80% moisture content, pumping is not technically possible due to high frictional losses (Septien et al., 2018).

To designate the service type allocation, the number of wet versus dry onsite facilities was estimated. Assuming that households with piped drinking water would most likely use water for flushing, JMP country coverage proportions for “Piped, Improved” drinking water were used as the proportions of ‘wet’ onsite sanitation facilities. Certain cultures use water for anal cleansing as opposed to wiping with supplies like toilet paper; however, country specific data on these cultural practices do not exist. This is a major assumption, and the number of ‘wet’ facilities may be underrepresented in areas where anal cleansing via washing is common regardless of the availability of piped, improved drinking water supply. Urban- and rural-specific piped water data was used when available; total country percentages for piped, improved water was used for countries lacking the urban and rural distinction. If JMP water facility type data were missing for a country, the urban/rural JMP service level percentages for “safely managed water” were used to indicate the number of ‘wet’ onsite facilities. Total country proportions for “safely managed” service levels were used for countries lacking the urban and rural distinction. The remaining pits were labeled ‘dry’ pits.

Assessments of the presence of trash and accessibility from the road and the resulting proportional impact on facilities that can be emptied were made based on a survey of 12 sanitation service providers via a series of key informant interviews conducted in 2018. This data is biased to East and Southern Africa and was primarily provided by vacuum truck operators working in urban centers. These service providers estimated that 75% of existing wet sanitation facilities can be emptied by conventional mechanized means, and 25% was inaccessible due to issues of trash and physical access. These proportions were extrapolated to all study areas, including Latin America and Asia, which may underestimate the number of mechanized emptiable facilities due to higher coverage of solid waste management services in Latin America (Grau et al., 2015) and Asia (Hondo et al., 2020), and potential differences in physical accessibility of onsite facilities.

#### 2.2.1 Service types

To better understand emptying service operators working in the onsite sanitation market, three Emptying Service Types were identified, with facility types allocated as per Fig. 1. Services are broadly categorized as facilities that are emptiable, via Mechanized or Non-Mechanized means, or Unemptiable.

The purpose of identifying these service types is to highlight needs required to shift to safely managed sanitation. Improved facilities are considered to be ‘Emptiable’ (sludge is able to be removed) due to the higher quality of the structure; the facilities can be emptied via Mechanized (by machine or machine-like practices, without risks of structure collapse), or Non-Mechanized (predominantly manual practices) means. Unimproved facilities require facility upgrades (which may range from basic upgrades such as adding a cement slab, to full super or sub structure replacement) and are considered Unemptiable (Table 1).

**Table 1:** Three current service types for onsite sanitation service providers and the assumptions used to calculate the Service Types. Examples for the types of equipment used in each Service Type are provided.

Service Type	Description	Servicing Equipment	Facility Types	Assumptions
*Mechanized*	Facilities that can readily be served by machine or ‘machine-like’ equipment	Vacuum Trucks Trash Pumps Innovative Technologies Gulper, Rammer, ROM, e-Vac, Flexcrevator, Excluder	75% of improved, wet facilities	75% of wet improved sanitation facilities can be emptied with existing equipment 25% of wet facilities cannot be serviced due to a high volume of trash in pit latrines, and the inability to access the pit due to density of housing or poor roads
*Non-Mechanized*	Remaining wet pits and all dry sanitation facilities that are of improved quality but are not accessible to mechanized emptying due to accessibility constraints or trash in the pit latrine	Buckets, shovels, garden tools, jerrycans and grabbing hooks	25% of improved, wet sanitation facilities 100% of improved, dry sanitation facilities	All improved facilities are robust enough to be serviced or emptied if emptying equipment can be adapted There is a market in servicing rural, improved facilities
*Unemptiable*	All unimproved facilities that need to be upgraded, due to the low quality of the super or sub structure, making emptying unsafe or impossible. Some facilities may be serviced with facility upgrades	N/A	100% of unimproved sanitation facilities	Some proportion of unimproved facilities can be upgraded, and others need to be rebuilt. No differentiation made between these two scenarios.

The scale of onsite facility needs is considered with the household as the base unit of measurement. Other onsite facilities (e.g., schools, businesses, lodges, hospitals) were not considered in this work, and thus the estimates of ‘total’ onsite facilities is conservative.

Service Types are presented both in terms of the physical number of urban and rural onsite facilities (volume), and ‘Service Type Density’ of need. Country sanitation ‘Service Type Density’ was determined using Equation (2):

(2) $$\;Service\;Type\;Density=\frac{Population\;using\;the\;Service\;Type}{Total\;Country\;Population}$$

where the Total Country Population includes the population using sewers and resorting to open defecation. The Volume of a service type indicates its prevalence in terms of physical numbers, while the Density of a service type indicates its prevalence within a country as a proportion of the full country population. The volumes and service type densities for each country studied are detailed in the Supplementary Information.

## 3 Results and discussion

The results of this work are first considered on a global level, followed by a more detailed analysis of each service type.

### 3.1 Overview of onsite sanitation facilities

An analysis of the number of onsite sanitation facilities across six SDG regions (Table 2) from 2017 JMP data shows that globally, over half of the considered LMIC population uses onsite sanitation facilities, totalling approximately 722 million onsite facilities. Of these, 590 million are improved, and 132 million are unimproved; 274 million onsite sanitation facilities are in urban areas, while 448 million are used in rural settings. However, improved facilities are almost ten times more numerous in urban than in rural areas. Onsite sanitation is often considered supplementary to sewers, serving areas where sewers cannot or do not reach (Orner and Mihelcic, 2017). However, this analysis shows that over half of the populations in four of the six SDG regions under analysis relies on onsite sanitation facilities, making these the dominant rather than the supplementary option. Improved facilities outnumber unimproved facilities by approximately 4:1 globally, though the ratio of improved to unimproved latrines varies widely throughout the regions. Improved latrines outnumber unimproved latrines 11:1 in Central and Southern Asia, but are almost equal in number in Sub Saharan Africa. This suggests that many households have invested in an onsite facility of a reasonable standard that should be emptied where possible.

Urban facilities typically need to be emptied due to space constraints which limit capacity for rebuilding new facilities. On the other hand, rural onsite sanitation facilities potentially can be safely decommissioned when full. However, a single cycle of decommissioning full pits will affect over 400 million latrines, serving a population of 2 billion. In this scenario, this population will rely on latrines being constructed-with the potential for slipping back temporarily, or even permanently, to open defecation. As such, all onsite facilities were considered as requiring sludge removal in this assessment. Though there is a need in both urban and rural settings, urban facilities can be considered an immediate need or market, and rural facilities considered an emerging market which needs further systems support to increase service reach and availability.

The countries with the most voluminous and dense use of onsite sanitation facilities from the studied SDG regions are shown in Table 3; results for all countries under analysis are provided in the Supplementary Information. These tables provide an overview of the presence of on-site sanitation facilities globally and can guide policy makers, product developers, NGOs, social enterprises and other organizations in targeting appropriate settings to trial and/or implementing new products, services, systems or programmes related to on-site sanitation. Organizations looking to maximize overall impact should target countries with a high volume and a high density of facilities. However, while volume and density provide a good starting point to target high-priority countries, a deeper understanding of the sanitation enabling environment is required before selecting target areas for work.

**Table 2:** The total number of 2017 household-level onsite sanitation facilities (in millions) per SDG region, including the density of onsite facilities calculated as the proportion of the population using onsite sanitation compared to the regions total population. Facilities are further distinguished and quantified as improved and unimproved.

SDG Region	No. of Onsite Facilities (millions)	Density of Onsite Facilities (%)	No. of Improved Facilities (millions)	No. of Unimproved Facilities (millions)	Ratio Improved: Unimproved
Central and Southern Asia	245	68%	225	20	11:1
Eastern and Southeastern Asia	273	74%	228	45	5:1
Latin America and the Caribbean	53	31%	43	10	4:1
Northern Africa and Western Asia	31	34%	26	4	6:1
Oceania	1	74%	0	1	0:1
Sub Saharan Africa	118	72%	67	52	1:1
**Total**	**722**	**56%**	**590**	**132**	**4:1**

**Table 3:** Top 10 countries with the most voluminous and dense markets from the studied SDG regions. The number of facilities accounts for the physical number of onsite units calculated from 2017 JMP data. The onsite sanitation density represents the percent of the country's population using onsite sanitation.

	Onsite Facilities by Volume			Onsite Facilities by Density		
Rank	Country	No. of Facilities	Onsite Sanitation Density	Country	Onsite Sanitation Density	No. of Facilities
1	India	166,156,772	64%	Tonga	100%	21,396
2	China	149,606,867	38%	Samoa	100%	39,062
3	Indonesia	47,239,997	79%	Anguilla	98%	3946
4	Bangladesh	23,962,487	95%	Guyana	97%	184,974
5	Vietnam	23,536,075	96%	Burundi	97%	1,921,992
6	Nigeria	21,508,675	70%	Rwanda	97%	2,455,882
7	Brazil	20,408,719	32%	Gambia	96%	181,291
8	Philippines	17,878,050	91%	Vietnam	96%	23,536,075
9	Thailand	16,942,805	91%	Suriname	96%	126,851
10	Pakistan	16,497,691	64%	Sri Lanka	95%	3,766,066

### 3.2 Service type overview

**Fig. 2:**
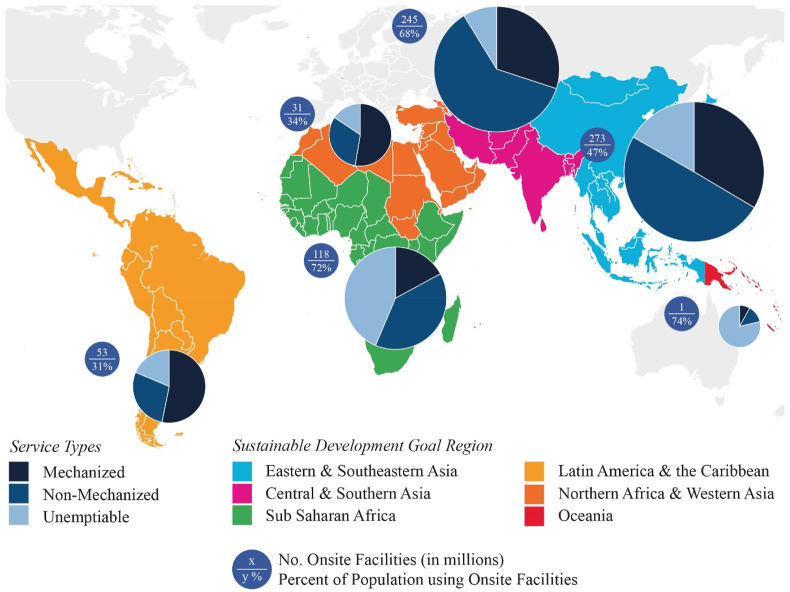
The proportion of Service Types of onsite sanitation facilities in six SDG regions of interest. The estimated 2017 number of household onsite facilities is displayed in millions. The percent of the population using onsite facilities is out of the SDG regions entire population, including sewer users and open defecators.

The Emptying Service Types presented in this paper are Mechanized (32% of facilities), Non-Mechanized (50%) and Unemptiable (18%) (Fig. 2). In this service typology, both Mechanized and Non-Mechanized categories can be emptied, whereas Unemptiable facilities need either upgrades or replacements to be emptiable. The goal should be for all onsite facilities to be emptied via Mechanized means, and the service types proposed act as a de-facto emptying service ladder, with Mechanized Emptying providing the preferred approach for both the household and the service provider due to increased speed, safety, and hygiene. It should be noted that while mechanized emptying enhances the safety of the emptying process, emptying is only one segment along the sanitation value chain and waste must be transported, treated, and reused or disposed before being considered safely managed. Fig. 2 shows the breakdown of emptying service types regionally.

While Oceania and Sub Saharan Africa demonstrate a substantial regional need for upgrading containment facilities based on the prevalence of Unemptiable facilities, the other SDG regions (and also Sub Saharan Africa) exhibit vast emptying needs. The high volume and prevalence of the Non-Mechanized emptying markets observed in Eastern and Southern Asia, Central and Southern Asia, and Sub Saharan Africa should make it a primary market focus, due to the lack of innovation in the systems, policies, regulation, technologies, and funding available for this emptying service level.

#### 3.2.1 Mechanized emptying

**Fig. 3:**
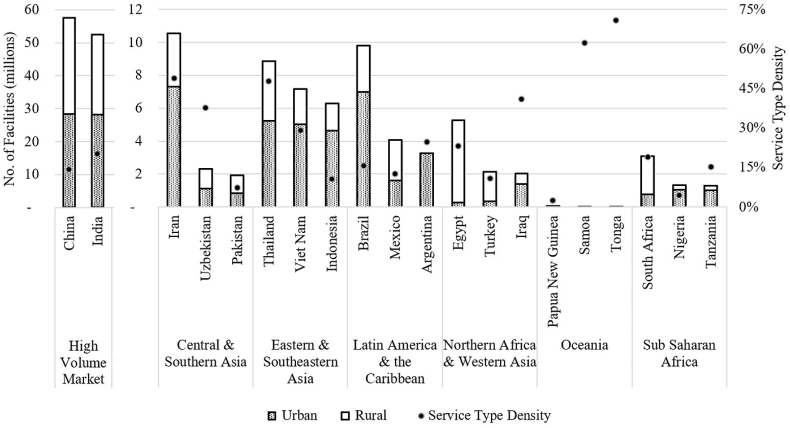
The top three countries per SDG region by number of facilities in the Mechanized Service Type. Country data is displayed for urban and rural facilities. The Service Type Density (filled dots, calculated according to Equation (2)) is calculated by dividing the Mechanized Service population over the total country population.

The Mechanized Emptying Service Type consists of facilities that can be emptied by a machine, or by a process aided by machinery, e.g., a manually operated pumping mechanism(Chipeta et al., 2017). The three countries in each region with the highest volume of onsite facilities that can be served by mechanized emptying are shown in Fig. 3, along with the country's mechanized service type density. India and China are presented separately as high-volume markets due to their high number of facilities compared to the rest of the world.

The urban market dominates this Mechanized service level, which is comprised of all septic tanks and wet, accessible, low-trash improved latrines. Globally, septic tanks contribute to the Mechanized market in urban (52%) and rural (48%) settings almost equally. The same trend is observed for three regions under analysis but not in Latin America and the Caribbean (63% of septic tanks in urban areas), Northern Africa and Western Asia (28% of septic tanks in urban areas) and Sub Saharan Africa (83% of septic tanks in urban areas). In this study, access to improved water sources is used as an assumption to define ‘wet’ facilities, which are more globally dominant in urban settings compared to rural (82% and 60% population coverage, respectively). This trend for more accessible flush water in urban settings could account for the higher number of urban compared to rural Mechanized facilities. It is important to note this assumption, which does not account for cultural practices like anal cleansing with water. Thus, the Mechanized market may be underestimated in this study, particularly in rural areas of Asia where anal washing is the preferred cleansing habit, or in religious countries which emphasize water cleansing practices.

The higher numbers in the urban Mechanized market suit the overall practicality of Mechanized emptying. Mechanized emptying involves public or private industry entrepreneurs who run and operate the business of vacuum trucks/pumps that service private households (Larsen et al., 2013). Higher capital investments are required for these types of businesses and may require more skilled operators. In urban centers, the potential for greater density of facilities in smaller areas and customer clustering, combined with the speed associated with servicing jobs with mechanized equipment, make the Mechanized market a potentially lucrative business, although financing mechanisms for purchasing Mechanized equipment may be required ([Bibr bib19][Bibr bib19]; Rao et al., 2016; Tillett et al., 2018).

The ‘improved’ and wet nature of onsite facilities being emptied by Mechanized methods means that developments required for this service type center around advances to the vacuum truck itself (ex. mobile treatment units) and other means(Sisco et al., 2017) of mechanically conveying, dewatering, treating, and reusing sludge. In addition, developments are required in business models, which incorporate smart technology to cluster customers and improve transport efficiency, public private partnerships to guarantee service coverage, service affordability, and waste treatment.

In a study which created a combined Shit Flow Diagram for 39 cities globally, 51% of the human waste generated came from onsite facilities; however, only 10% of this waste was contained and emptied and only 5% made it to a treatment facility (Peal et al., 2020). While the Mechanized emptying service level allows waste to be safely emptied and transported, it does not guarantee sludge will be safely treated. Funding for treatment facilities and capacity building for monitored operation of the facilities, coupled with policies and regulations to ensure transported sludge arrives at treatment facilities, and receives adequate treatment prior to discharge or reuse, are all required to ensure safely managed sanitation.

In addition, parallel infrastructure development including menstrual hygiene and solid waste management, road access, water accessibility, and decentralized faecal sludge treatment or transfer facilities would increase the number of onsite facilities accessible to Mechanized emptying. This can elevate a large portion of the Non-Mechanized households to Mechanized emptying, improving the likelihood of sludge being safely managed.

#### 3.2.2 Non-mechanized emptying

**Fig. 4:**
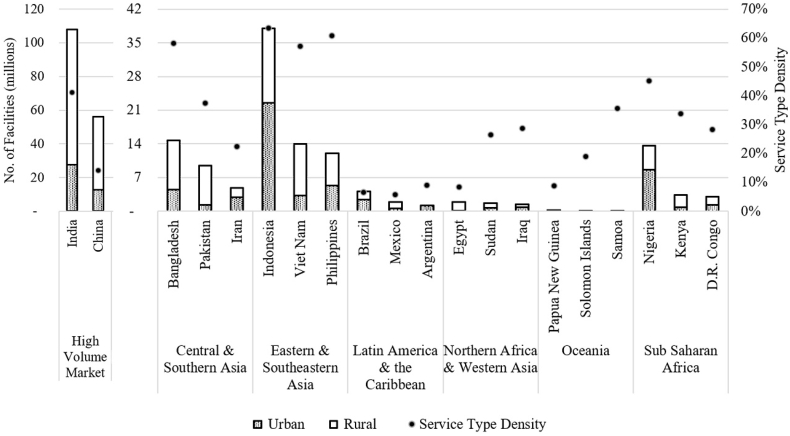
The top three countries per SDG region by number of facilities in the Non-Mechanized Service Type. Country data is displayed for urban and rural facilities. The Service Type Density (filled dots) is calculated by dividing the Non-Mechanized Service population over the total country population.

Non-Mechanized emptying exists in countries with dry, inaccessible facilities, or facilities that may have trash, preventing their emptying using conventional means. Non-Mechanized facilities are emptied by using buckets, shovels, and garden tools, and are often performed by informal businesses that indiscriminately dispose of waste. Improvements in this emptying servicing segment represent an urgent area of intervention. The Non-Mechanized service type is the most voluminous market in the SDG regions under analysis (Fig. 4), outnumbering (2:1 ratio) the number of facilities in the Mechanized service type. The Non-Mechanized service type is dominant in terms of volume and density in Eastern and Southeastern Asia; a high density of need is also seen in Sub Saharan Africa.

Comparing the top three countries in the Non-Mechanized market (Fig. 4) with the Mechanized market (Fig. 3) shows that the higher volume of Non-Mechanized facilities are coupled with a higher density as well. The combination of high volume and high density, especially in India, Indonesia, Bangladesh, Vietnam, the Philippines and Nigeria, could provide system/program/technology innovators with a penetrable and voluminous market to test new ideas or technologies with the goal of pushing more households out of the Non-Mechanized emptying type and into the Mechanized servicing level.

Technical innovations for the Non-Mechanized market should focus on enabling the facility to be emptied via Mechanized means. For the facility itself, improvements can be made via: restricting solid waste that enters the latrine, accompanied with user behaviour change, increasing access to water, improving pit lining, and installing low-volume flush toilets to reduce the viscosity of the contents to allow pumping. Developments required in this space include: local manufacture of smaller, more affordable vacuum trucks to ease market entry for service providers and improve access to dense urban areas, and add-ons to trucks that screen trash and protect the pumps. In rural areas, the same technical innovations are required, as well as significant systems innovation to enable an affordable service to reach customers in areas far from treatment infrastructure.

In practice, providing rural areas with improved, mobile latrine superstructures, building capacity for digging and lining pits, and creating effective slabs so new pits can be constructed when old ones are filled, may be more effective strategies than converting rural Non-Mechanized customers to Mechanized, since space for new latrines may not be limiting in rural environments.

In the absence of regulations, safety standards, or customers' ability to pay, it is difficult to shift the basic service in this segment away from that being offered by some men with buckets. Ability to pay is a challenge in this segment, as many of the households that are hardest to reach are in dense low-income urban settlements. However, in the majority of these settlements, informal services and a culture of paying for sanitation services already exist. With improved safety standards, enforced regulations, and/or incentives for users of improved services, there is a large theoretical demand for improved emptying services. We estimate that over 300 million facilities are of this service type in the SDG regions considered, and it is important to boost intensive research and development to safely manage sludge and improve sanitation workers’ practices.

#### 3.2.3 Unemptiable

Unemptiable facilities are those judged to not be physically emptiable via mechanized or manual means due to a lack of structural integrity. All unimproved facilities (e.g., pits lacking a true slab) are Unemptiable for the purposes of this study, whether wet or dry. Continuing to build Unemptiable latrines puts sustainable access to safely managed sanitation at risk. Developments in technical, systems, and financial systems are required to enable safe disposal of waste in-situ without the need for recurring rebuilding.

**Fig. 5:**
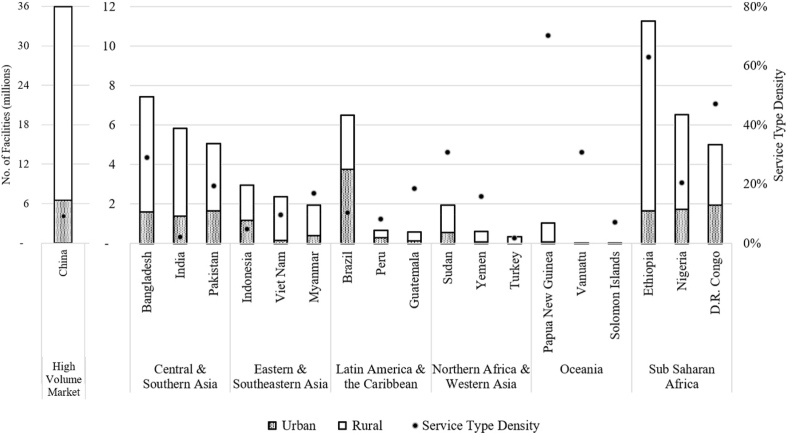
The top three countries per SDG region by number of facilities in the Unemptiable Service Type. Country data is displayed for urban and rural facilities. The Service Type Density (filled dots) is calculated by dividing the Unemptiable Service population over the total country population.

Globally, China and Ethiopia have the most Unemptiable pits, while Sub Saharan Africa exhibits the highest regional prevalence of this service type (Fig. 5). In terms of facility density, Ethiopia, Nigeria, and the Democratic Republic of Congo have high prevalence of Unemptiable latrines and subsequent need for facility rebuilds or upgrades.

Analysis of Unemptiable facilities shifts focus to rural areas, where unimproved facilities dominate. In this segment, customers may be disperse, and thus it is particularly important in this category to consider the overall market density. For these Unemptiable pits, the technical needs center around the pit itself, in the form of replacements or upgrades of the current structure. Many facilities would likely need to be decommissioned onsite. There is an opportunity in low cost but emptiable latrines to replace those that are decommissioned, or in sanitation upgrades to enhance the safety or usability of the systems in place. These upgrades include removable and reusable superstructures once a pit is full and decommissioned. This market can be ideal for upgrade products for sanitation facilities such as the SaTo pan (manufactured by Lixil). However, it has been stated that the potential for private sector approaches to upgrades or new facility options in the Unemptiable Service Type is less certain than those for emptying (Capone et al., 2020). In addition, households with unimproved facilities may be unaccustomed to these products or services, and ability or willingness to pay may be low.

Sustainable rural sanitation needs to include educating entire communities on the benefits of well managed sanitation so that communities maintain functioning facilities (Mukherjee et al., 2009; O'Reilly and Louis, 2014; Perez et al., 2012). Community involvement, coupled with sanitation and hygiene promotional activities encourage long-term sustainable behaviour change (Perez et al., 2012). Capacity building for local masons and entrepreneurs, coupled with accreditation and sanitation marketing and availability of a selection of sanitation products, increases consumer trust, demand, and adoption for improved facilities, leading to more sustained safely managed sanitation in rural communities (Mukherjee et al., 2009; Perez et al., 2012).

While rural latrines may become physically emptiable (via mechanized or non-mechanized means) via upgrades, building sufficient accessible treatment infrastructure to serve the needs of sludge removed from these facilities will remain a challenge; in the short-term, building systems that remain sustainable while minimizing the need for sludge removal may be more strategic.

## 4 Conclusions

This study provides high-level market insights for governments, donors, non-government organizations (NGOs), and sanitation entrepreneurs considering market-based approaches to improving systems, facilities, or servicing of onsite sanitation facilities in six SDG regions. Three service typologies are proposed based primarily on technical factors: Emptiable facilities – consisting of both Mechanized and Non-Mechanized options, and Unemptiable facilities.

The data shows that while improved sanitation is reaching many onsite sanitation facility owners, there is potential to reach the 50% of onsite sanitation users whose facilities are currently emptied manually. Mechanized Emptying technologies or systems to empty dry improved pits should be an area of intensive, targeted, and in-depth research. Similarly, the lifetimes of rural latrines may be short without system innovations that provide affordable options for servicing, replacing, or emptying pits that are far from treatment infrastructure.

Overall, the onsite sanitation market holds significant market opportunity and the potential to have a high impact to improve the health and dignity of users and service providers of onsite sanitation facilities. This study provides regional and country level market data, which can be used in more detailed market analysis to stimulate both the public and private sectors to address the needs of populations served by onsite sanitation with a goal of achieving safely managed sanitation.

## Funding acquisition

Acquisition of the financial support for the project leading to this publication.

## Author contributions

Nicola Greene: Conceptualization, Methodology, Formal analysis, Investigation, Writing – original draft, Visualization. Sarah Hennessy: Formal analysis, Investigation, Writing – original draft, Visualization. Tate W. Rogers: Conceptualization, Writing – review & editing. Jocelyn Tsai: Writing – review & editing, Supervision, Project administration. Francis de los Reyes III: Conceptualization, Writing – review & editing, Supervision, Funding acquisition

## Funding sources

This work was supported by the 10.13039/100000865Bill & Melinda Gates Foundation, Seattle, WA [OPP1094923].

## Declaration of competing interest

The authors declare that they have no known competing financial interests or personal relationships that could have appeared to influence the work reported in this paper.
