# Supplementary material for: The role of emptying services in provision of safely managed sanitation: A classification and quantification of the needs of LMICs
Source: J Environ Manage. 2021 Jul 15;290:112612. doi: 10.1016/j.jenvman.2021.112612 (PMC8178437; doi:10.1016/j.jenvman.2021.112612)
Supplement: Multimedia component 1 [file mmc1.docx]

**Supplementary Information**

**The role of emptying services in provision of safely managed sanitation: a classification and quantification of the needs of LMICs**

Nicola Greene^1^, Sarah Hennessy^1^, Tate W. Rogers^1^, Jocelyn Tsai^2^, and Francis L. de los Reyes III*^2^

^1^Triangle Environmental Health Initiative, LLC, Durham, NC, USA

^2^Department of Civil, Construction, and Environmental Engineering, North Carolina State University, Raleigh, NC USA

Table 1. The number of onsite sanitation facilities alphabetized by country per Service Type. The Service Type Density is calculated by dividing the population using the specified service by the total country population. Thus, the Service Type Density covers users of sewers, onsite facilities, and open defecators. The number of facilities required for Open Defecators is estimated assuming the facilities will not be shared between households. Urban and rural proportions of the onsite facilities are provided. “ND” indicates no data available from JMP.

|  | **Mechanized** | | | | **Non-Mechanized** | | | | **Unemptiable** | | | | **Open Defecation** | | | |
| --- | --- | --- | --- | --- | --- | --- | --- | --- | --- | --- | --- | --- | --- | --- | --- | --- |
| **Country** | **No. of Facilities** | **Service Type Density** | **Urban** | **Rural** | **No. of Facilities** | **Service Type Density** | **Urban** | **Rural** | **No. of Facilities** | **Service Type Density** | **Urban** | **Rural** | **No. of Facilities**  **Required** | **Service Type Density** | **Urban** | **Rural** |
| Afghanistan | 367,345 | 10% | 66% | 34% | 1,599,720 | 41% | 31% | 69% | 1,351,493 | 34% | 12% | 88% | 563,238 | 13% | 0% | 100% |
| Algeria | 482,751 | 6% | 27% | 73% | 465,887 | 6% | 18% | 82% | 255,876 | 3% | 63% | 37% | 64,417 | 1% | 18% | 82% |
| Angola | 1,054,451 | 21% | 97% | 3% | 1,817,690 | 36% | 75% | 25% | 516,612 | 10% | 44% | 56% | 1,232,071 | 20% | 3% | 97% |
| Anguilla | 2,875 | 72% | 100% | 0% | 1,056 | 26% | 100% | 0% | 15 | 0% | 100% | 0% | 22 | 1% | 100% | 0% |
| Argentina | 3,266,830 | 25% | 100% | 0% | 1,178,771 | 9% | 100% | 0% | ND | ND | ND | ND | 215,594 | 2% | 100% | 0% |
| Armenia | 129,305 | 18% | 2% | 98% | 46,930 | 6% | 2% | 98% | 42,431 | 6% | 3% | 97% | ND | ND | ND | ND |
| Azerbaijan | 710,244 | 33% | 41% | 59% | 477,267 | 22% | 22% | 78% | 100,844 | 5% | 0% | 100% | 3,357 | 0% | 0% | 100% |
| Bangladesh | 1,809,175 | 8% | 90% | 10% | 14,713,956 | 58% | 30% | 70% | 7,439,355 | 29% | 22% | 78% | ND | ND | ND | ND |
| Belize | 53,989 | 56% | 46% | 54% | 30,453 | 32% | 33% | 67% | 2,199 | 2% | 10% | 90% | 804 | 1% | 44% | 56% |
| Benin | 177,316 | 12% | 86% | 14% | 320,076 | 22% | 70% | 30% | 147,124 | 10% | 55% | 45% | 1,212,634 | 54% | 25% | 75% |
| Bhutan | 78,556 | 54% | 41% | 59% | 26,462 | 18% | 42% | 58% | 31,770 | 22% | 23% | 77% | ND | ND | ND | ND |
| Bolivia (Plurinational State of) | 401,711 | 15% | 81% | 19% | 408,256 | 15% | 42% | 58% | 240,403 | 9% | 31% | 69% | 416,246 | 13% | 11% | 89% |
| Botswana | 350,499 | 56% | 81% | 19% | 155,396 | 25% | 68% | 32% | 37,746 | 6% | 67% | 33% | 71,751 | 11% | 7% | 93% |
| Brazil | 9,820,972 | 16% | 71% | 29% | 4,085,672 | 6% | 58% | 42% | 6,502,075 | 10% | 58% | 42% | 711,618 | 1% | 13% | 87% |
| Burkina Faso | 297,668 | 15% | 94% | 6% | 611,433 | 31% | 35% | 65% | 124,157 | 6% | 20% | 80% | 1,511,812 | 47% | 5% | 95% |
| Burundi | 301,796 | 17% | 42% | 58% | 809,089 | 40% | 8% | 92% | 811,107 | 40% | 5% | 95% | 58,964 | 3% | 3% | 97% |
| CÃ´te d'Ivoire | 641,970 | 17% | 86% | 14% | 1,103,284 | 30% | 56% | 44% | 710,823 | 20% | 41% | 59% | 1,290,804 | 26% | 16% | 84% |
| Cabo Verde | 39,459 | 37% | 66% | 34% | 20,771 | 19% | 50% | 50% | ND | ND | ND | ND | 22,749 | 20% | 40% | 60% |
| Cambodia | 324,580 | 10% | 67% | 33% | 1,327,866 | 42% | 11% | 89% | 81,037 | 3% | 0% | 100% | 1,101,855 | 32% | 0% | 100% |
| Cameroon | 833,149 | 22% | 95% | 5% | 1,334,099 | 35% | 71% | 29% | 1,349,267 | 35% | 24% | 76% | 334,942 | 7% | 11% | 89% |
| Chad | 90,958 | 5% | 97% | 3% | 176,746 | 10% | 77% | 23% | 327,794 | 18% | 35% | 65% | 1,727,729 | 67% | 6% | 94% |
| Chile | 434,396 | 9% | 14% | 86% | 150,128 | 3% | 14% | 86% | ND | ND | ND | ND | ND | ND | ND | ND |
| China | 57,659,216 | 14% | 49% | 51% | 56,011,249 | 14% | 23% | 77% | 35,936,402 | 9% | 18% | 82% | 942,735 | 0% | 16% | 84% |
| China, Hong Kong Special Administrative Region | 67,661 | 3% | 100% | 0% | 22,554 | 1% | 100% | 0% | 90,215 | 4% | 100% | 0% | ND | ND | ND | ND |
| Colombia | 1,241,275 | 9% | 34% | 66% | 1,098,937 | 8% | 16% | 84% | 298,969 | 2% | 35% | 65% | 426,593 | 3% | 20% | 80% |
| Comoros | 24,876 | 20% | 44% | 56% | 29,541 | 24% | 28% | 72% | 62,741 | 50% | 21% | 79% | 896 | 1% | 26% | 74% |
| Congo | 187,327 | 26% | 99% | 1% | 187,252 | 26% | 83% | 17% | 272,074 | 37% | 44% | 56% | 106,806 | 9% | 12% | 88% |
| Costa Rica | 796,213 | 56% | 74% | 26% | 268,139 | 19% | 75% | 25% | 20,688 | 1% | 48% | 52% | 2,885 | 0% | 57% | 43% |
| Cuba | 923,966 | 26% | 69% | 31% | 730,260 | 20% | 48% | 52% | 139,880 | 4% | 73% | 27% | 9,841 | 0% | 46% | 54% |
| Cyprus | 152,060 | 35% | 37% | 63% | 51,355 | 12% | 38% | 62% | 3,640 | 1% | 37% | 63% | ND | ND | ND | ND |
| Democratic People's Republic of Korea | 1,186,501 | 18% | 47% | 53% | 1,350,653 | 21% | 31% | 69% | 999,525 | 16% | 32% | 68% | ND | ND | ND | ND |
| Democratic Republic of the Congo | 1,190,683 | 12% | 92% | 8% | 2,977,453 | 28% | 45% | 55% | 5,014,610 | 47% | 39% | 61% | 1,849,024 | 12% | 13% | 87% |
| Djibouti | 83,605 | 45% | 98% | 2% | 36,798 | 20% | 80% | 20% | 23,384 | 13% | 79% | 21% | 34,213 | 17% | 14% | 86% |
| Dominican Republic | 1,244,050 | 44% | 81% | 19% | 826,656 | 29% | 71% | 29% | 60,474 | 2% | 68% | 32% | 88,993 | 3% | 52% | 48% |
| Ecuador | 808,175 | 20% | 37% | 63% | 568,622 | 14% | 19% | 81% | 34,834 | 1% | 0% | 100% | 92,213 | 2% | 0% | 100% |
| Egypt | 5,253,124 | 23% | 5% | 95% | 1,894,754 | 8% | 5% | 95% | 329,087 | 1% | 5% | 95% | ND | ND | ND | ND |
| El Salvador | 539,932 | 38% | 58% | 42% | 288,237 | 20% | 44% | 56% | 9,792 | 1% | 25% | 75% | 15,759 | 1% | 0% | 100% |
| Equatorial Guinea | 48,946 | 21% | 87% | 13% | 107,886 | 45% | 70% | 30% | 50,416 | 21% | 55% | 45% | 7,469 | 3% | 64% | 36% |
| Eswatini | 74,777 | 33% | 30% | 70% | 98,097 | 42% | 10% | 90% | 18,333 | 8% | 17% | 83% | 20,311 | 7% | 2% | 98% |
| Ethiopia | 973,825 | 7% | 90% | 10% | 1,065,109 | 7% | 50% | 50% | 11,264,896 | 63% | 15% | 85% | 5,083,519 | 22% | 5% | 95% |
| Gabon | 99,362 | 27% | 97% | 3% | 55,087 | 15% | 73% | 27% | 83,884 | 22% | 79% | 21% | 14,595 | 3% | 76% | 24% |
| Gambia | 66,628 | 36% | 81% | 19% | 51,896 | 28% | 62% | 38% | 62,766 | 33% | 36% | 64% | 2,749 | 1% | 0% | 100% |
| Georgia | 212,730 | 19% | 25% | 75% | 223,258 | 20% | 9% | 91% | 98,649 | 9% | 20% | 80% | ND | ND | ND | ND |
| Ghana | 718,851 | 17% | 77% | 23% | 2,047,236 | 49% | 63% | 37% | 551,371 | 13% | 35% | 65% | 1,490,542 | 18% | 23% | 77% |
| Guatemala | 608,837 | 19% | 34% | 66% | 479,195 | 15% | 19% | 81% | 579,187 | 19% | 21% | 79% | 164,961 | 5% | 11% | 89% |
| Guinea | 187,262 | 15% | 92% | 8% | 455,459 | 36% | 42% | 58% | 427,418 | 33% | 15% | 85% | 291,966 | 14% | 3% | 97% |
| Guinea-Bissau | 52,469 | 19% | 85% | 15% | 38,086 | 14% | 69% | 31% | 131,395 | 47% | 29% | 71% | 64,810 | 17% | 4% | 96% |
| Guyana | 87,388 | 46% | 34% | 66% | 91,191 | 48% | 17% | 83% | 6,395 | 3% | 18% | 82% | 1,255 | 1% | 0% | 100% |
| Haiti | 177,166 | 10% | 67% | 33% | 898,125 | 51% | 71% | 29% | 322,089 | 18% | 33% | 67% | 492,899 | 20% | 23% | 77% |
| Honduras | 634,223 | 33% | 36% | 64% | 338,788 | 17% | 26% | 74% | 73,273 | 4% | 40% | 60% | 124,620 | 6% | 18% | 82% |
| India | 52,459,682 | 20% | 54% | 46% | 107,852,308 | 41% | 26% | 74% | 5,844,783 | 2% | 24% | 76% | 75,365,587 | 26% | 6% | 94% |
| Indonesia | 6,299,994 | 11% | 73% | 27% | 37,989,342 | 64% | 59% | 41% | 2,950,662 | 5% | 40% | 60% | 6,398,015 | 10% | 22% | 78% |
| Iran (Islamic Republic of) | 10,546,023 | 49% | 69% | 31% | 4,819,563 | 22% | 59% | 41% | 391,067 | 2% | 41% | 59% | 22,326 | 0% | 100% | 0% |
| Iraq | 2,020,541 | 41% | 69% | 31% | 1,409,704 | 29% | 57% | 43% | 233,366 | 5% | 35% | 65% | 990 | 0% | 100% | 0% |
| Israel | 16,783 | 1% | 55% | 45% | 5,594 | 0% | 55% | 45% | ND | ND | ND | ND | ND | ND | ND | ND |
| Jamaica | 407,614 | 47% | 52% | 48% | 257,729 | 29% | 36% | 64% | 4,446 | 1% | 56% | 44% | 4,956 | 1% | 100% | 0% |
| Jordan | 419,453 | 22% | 79% | 21% | 227,180 | 12% | 73% | 27% | 25,741 | 1% | 87% | 13% | 3,688 | 0% | 55% | 45% |
| Kazakhstan | 1,777,876 | 35% | 43% | 57% | 1,453,857 | 28% | 22% | 78% | 4,818 | 0% | 52% | 48% | ND | ND | ND | ND |
| Kenya | 1,127,909 | 12% | 60% | 40% | 3,385,044 | 34% | 25% | 75% | 3,949,160 | 38% | 13% | 87% | 1,412,936 | 10% | 5% | 95% |
| Kyrgyzstan | 800,981 | 57% | 29% | 71% | 415,294 | 29% | 20% | 80% | 1,822 | 0% | 100% | 0% | ND | ND | ND | ND |
| Lao People's Democratic Republic | 536,682 | 31% | 66% | 34% | 785,397 | 45% | 28% | 72% | 35,984 | 2% | 10% | 90% | 368,066 | 21% | 2% | 98% |
| Lesotho | 157,384 | 31% | 51% | 49% | 166,100 | 30% | 26% | 74% | 58,485 | 10% | 14% | 86% | 182,142 | 27% | 6% | 94% |
| Liberia | 13,224 | 2% | 100% | 0% | 232,565 | 41% | 72% | 28% | 87,898 | 16% | 51% | 49% | 378,996 | 40% | 25% | 75% |
| Madagascar | 278,684 | 9% | 86% | 14% | 491,354 | 16% | 44% | 56% | 907,239 | 29% | 42% | 58% | 2,303,053 | 45% | 19% | 81% |
| Malawi | 222,735 | 7% | 71% | 29% | 980,159 | 30% | 11% | 89% | 1,767,452 | 55% | 12% | 88% | 235,971 | 6% | 4% | 96% |
| Malaysia | ND | ND | ND | ND | ND | ND | ND | ND | 29,696 | 0% | 24% | 76% | ND | ND | ND | ND |
| Maldives | 3,653 | 4% | 0% | 100% | 28,822 | 35% | 0% | 100% | ND | ND | ND | ND | ND | ND | ND | ND |
| Mali | 594,165 | 23% | 87% | 13% | 824,936 | 30% | 43% | 57% | 1,116,069 | 39% | 18% | 82% | 235,361 | 7% | 6% | 94% |
| Marshall Islands | 459 | 5% | 75% | 25% | 4,552 | 45% | 70% | 30% | 85 | 1% | 25% | 75% | 1,066 | 10% | 26% | 74% |
| Mauritania | 198,560 | 24% | 86% | 14% | 244,111 | 30% | 71% | 29% | 95,461 | 12% | 36% | 64% | 291,371 | 32% | 14% | 86% |
| Mauritius | 202,288 | 57% | 27% | 73% | 67,849 | 19% | 27% | 73% | 1,424 | 0% | 10% | 90% | 476 | 0% | 0% | 100% |
| Mexico | 4,068,822 | 12% | 40% | 60% | 1,849,795 | 6% | 31% | 69% | 425,667 | 1% | 25% | 75% | 305,635 | 1% | 27% | 73% |
| Mongolia | 70,643 | 13% | 94% | 6% | 293,320 | 53% | 64% | 36% | 17,685 | 3% | 54% | 46% | 73,432 | 10% | 7% | 93% |
| Morocco | 1,109,694 | 17% | 31% | 69% | 1,355,237 | 20% | 10% | 90% | 32,583 | 0% | 96% | 4% | 495,549 | 7% | 1% | 99% |
| Mozambique | 798,472 | 13% | 90% | 10% | 1,248,614 | 20% | 46% | 54% | 2,406,062 | 39% | 28% | 72% | 1,862,203 | 27% | 10% | 90% |
| Myanmar | 1,734,789 | 15% | 75% | 25% | 6,785,749 | 59% | 26% | 74% | 1,955,819 | 17% | 20% | 80% | 1,190,510 | 9% | 4% | 96% |
| Namibia | 31,058 | 6% | 39% | 61% | 27,022 | 5% | 17% | 83% | 22,089 | 4% | 45% | 55% | 291,225 | 49% | 23% | 77% |
| Nauru | 518 | 29% | 100% | 0% | 789 | 44% | 100% | 0% | 19 | 1% | 100% | 0% | 59 | 3% | 100% | 0% |
| Nepal | 1,528,268 | 25% | 23% | 77% | 2,777,906 | 45% | 18% | 82% | 170,769 | 3% | 7% | 93% | 1,485,547 | 21% | 7% | 93% |
| Nicaragua | 324,908 | 27% | 75% | 25% | 345,613 | 29% | 28% | 72% | 161,058 | 13% | 40% | 60% | 82,183 | 7% | 9% | 91% |
| Niger | 241,748 | 9% | 81% | 19% | 350,990 | 13% | 34% | 66% | 233,185 | 9% | 23% | 77% | 2,462,828 | 68% | 3% | 97% |
| Nigeria | 1,338,836 | 5% | 77% | 23% | 13,626,535 | 45% | 63% | 37% | 6,543,304 | 21% | 26% | 74% | 7,712,798 | 20% | 22% | 78% |
| Oman | 347,242 | 60% | 85% | 15% | 168,719 | 29% | 75% | 25% | ND | ND | ND | ND | ND | ND | ND | ND |
| Pakistan | 1,922,983 | 7% | 43% | 57% | 9,520,909 | 37% | 14% | 86% | 5,053,800 | 20% | 33% | 67% | 3,019,704 | 10% | 0% | 100% |
| Palau | 1,399 | 32% | 59% | 41% | 490 | 11% | 56% | 44% | ND | ND | ND | ND | ND | ND | ND | ND |
| Panama | 378,841 | 36% | 61% | 39% | 173,360 | 16% | 47% | 53% | 68,040 | 6% | 25% | 75% | 49,363 | 4% | 7% | 93% |
| Papua New Guinea | 36,215 | 2% | 72% | 28% | 130,960 | 9% | 28% | 72% | 1,036,647 | 70% | 8% | 92% | 238,196 | 14% | 1% | 99% |
| Paraguay | 849,591 | 59% | 63% | 37% | 361,021 | 25% | 55% | 45% | 89,659 | 6% | 11% | 89% | 9,731 | 1% | 42% | 58% |
| Peru | 659,564 | 8% | 40% | 60% | 498,951 | 6% | 24% | 76% | 659,943 | 8% | 45% | 55% | 559,737 | 7% | 36% | 64% |
| Philippines | 5,183,299 | 26% | 56% | 44% | 12,003,167 | 61% | 44% | 56% | 691,584 | 3% | 31% | 69% | 1,125,296 | 5% | 24% | 76% |
| Rwanda | 546,396 | 22% | 35% | 65% | 1,474,962 | 57% | 11% | 89% | 434,524 | 17% | 10% | 90% | 62,775 | 2% | 13% | 87% |
| Saint Lucia | 30,757 | 68% | 17% | 83% | 12,020 | 26% | 15% | 85% | 9 | 0% | 100% | 0% | 390 | 1% | 100% | 0% |
| Samoa | 24,443 | 62% | 20% | 80% | 13,943 | 36% | 16% | 84% | 677 | 2% | 15% | 85% | 41 | 0% | 16% | 84% |
| Sao Tome and Principe | 11,881 | 24% | 81% | 19% | 4,847 | 10% | 70% | 30% | 1,713 | 4% | 80% | 20% | 25,263 | 47% | 63% | 37% |
| Senegal | 542,443 | 34% | 65% | 35% | 419,999 | 26% | 47% | 53% | 294,206 | 18% | 18% | 82% | 262,473 | 14% | 6% | 94% |
| Sierra Leone | 76,174 | 11% | 80% | 20% | 265,699 | 38% | 57% | 43% | 220,352 | 33% | 25% | 75% | 225,757 | 18% | 14% | 86% |
| Solomon Islands | 14,703 | 13% | 67% | 33% | 21,007 | 19% | 42% | 58% | 8,253 | 7% | 0% | 100% | 65,488 | 54% | 2% | 98% |
| Somalia | 432,809 | 18% | 92% | 8% | 615,254 | 25% | 50% | 50% | 479,079 | 19% | 29% | 71% | 837,760 | 28% | 2% | 98% |
| South Africa | 3,086,040 | 19% | 24% | 76% | 2,173,798 | 13% | 12% | 88% | 1,283,620 | 8% | 30% | 70% | 252,761 | 1% | 31% | 69% |
| South Sudan | 12,070 | 1% | 81% | 19% | 280,499 | 18% | 54% | 46% | 269,050 | 18% | 36% | 64% | 1,328,107 | 63% | 4% | 96% |
| Sri Lanka | 1,051,510 | 27% | 32% | 68% | 2,691,203 | 68% | 10% | 90% | 23,354 | 1% | 91% | 9% | 22,590 | 1% | 0% | 100% |
| Sudan | 1,108,262 | 17% | 71% | 29% | 1,679,767 | 27% | 44% | 56% | 1,940,332 | 31% | 29% | 71% | 1,762,800 | 24% | 2% | 98% |
| Suriname | 74,000 | 56% | 76% | 24% | 50,011 | 38% | 58% | 42% | 2,840 | 2% | 34% | 66% | 4,018 | 3% | 8% | 92% |
| Syrian Arab Republic | 502,808 | 14% | 8% | 92% | 439,622 | 13% | 7% | 93% | 29,763 | 1% | 22% | 78% | ND | ND | ND | ND |
| Tajikistan | 532,981 | 38% | 22% | 78% | 656,926 | 47% | 8% | 92% | 7,035 | 1% | 17% | 83% | 938 | 0% | 0% | 100% |
| Thailand | 8,858,603 | 48% | 59% | 41% | 8,073,769 | 44% | 35% | 65% | 10,433 | 0% | 100% | 0% | ND | ND | ND | ND |
| Timor-Leste | 52,176 | 26% | 52% | 48% | 50,629 | 25% | 33% | 67% | 36,111 | 18% | 15% | 85% | 43,282 | 20% | 0% | 100% |
| Togo | 117,191 | 12% | 93% | 7% | 307,702 | 30% | 72% | 28% | 102,588 | 10% | 29% | 71% | 817,072 | 48% | 11% | 89% |
| Tonga | 15,145 | 71% | 24% | 76% | 5,068 | 24% | 24% | 76% | 1,183 | 6% | 11% | 89% | ND | ND | ND | ND |
| Tunisia | 545,423 | 25% | 39% | 61% | 312,828 | 14% | 23% | 77% | 89,688 | 4% | 41% | 59% | ND | ND | ND | ND |
| Turkey | 2,136,881 | 11% | 17% | 83% | 727,803 | 4% | 17% | 83% | 353,119 | 2% | 0% | 100% | 62,826 | 0% | 0% | 100% |
| Turkmenistan | 289,678 | 26% | 52% | 48% | 499,017 | 45% | 22% | 78% | 304 | 0% | 0% | 100% | ND | ND | ND | ND |
| Tuvalu | 279 | 13% | 39% | 61% | 97 | 5% | 38% | 62% | 31 | 1% | 100% | 0% | 157 | 7% | 51% | 49% |
| Uganda | 462,116 | 8% | 78% | 22% | 1,801,767 | 28% | 33% | 67% | 3,965,060 | 58% | 12% | 88% | 531,603 | 6% | 8% | 92% |
| United Republic of Tanzania | 1,309,227 | 15% | 77% | 23% | 2,841,520 | 31% | 48% | 52% | 4,128,309 | 41% | 13% | 87% | 1,387,845 | 12% | 5% | 95% |
| Uruguay | 354,925 | 29% | 88% | 12% | 119,252 | 10% | 88% | 12% | 8,197 | 1% | 88% | 12% | 4,954 | 0% | 99% | 1% |
| Uzbekistan | 2,297,007 | 38% | 49% | 51% | 2,408,844 | 40% | 24% | 76% | ND | ND | ND | ND | ND | ND | ND | ND |
| Vanuatu | 8,028 | 22% | 52% | 48% | 12,681 | 35% | 23% | 77% | 11,149 | 31% | 6% | 94% | 304 | 1% | 20% | 80% |
| Viet Nam | 7,181,784 | 29% | 70% | 30% | 13,980,443 | 57% | 23% | 77% | 2,373,847 | 10% | 7% | 93% | 754,923 | 3% | 16% | 84% |
| Wallis and Futuna Islands | ND | ND | ND | ND | ND | ND | ND | ND | ND | ND | ND | ND | 21 | 1% | 0% | 100% |
| West Bank and Gaza Strip | 221,101 | 23% | 39% | 61% | 249,137 | 26% | 69% | 31% | ND | ND | ND | ND | 1,624 | 0% | 0% | 100% |
| Yemen | 454,584 | 11% | 44% | 56% | 937,902 | 24% | 15% | 85% | 623,621 | 16% | 13% | 87% | 828,691 | 20% | 2% | 98% |
| Zambia | 256,322 | 11% | 96% | 4% | 608,222 | 24% | 43% | 57% | 964,024 | 37% | 32% | 68% | 643,109 | 19% | 6% | 94% |
| Zimbabwe | 172,661 | 6% | 59% | 41% | 990,920 | 32% | 9% | 91% | 330,921 | 11% | 11% | 89% | 1,010,754 | 25% | 0% | 100% |

**Joint Monitoring Programme Service Levels for Sanitation**


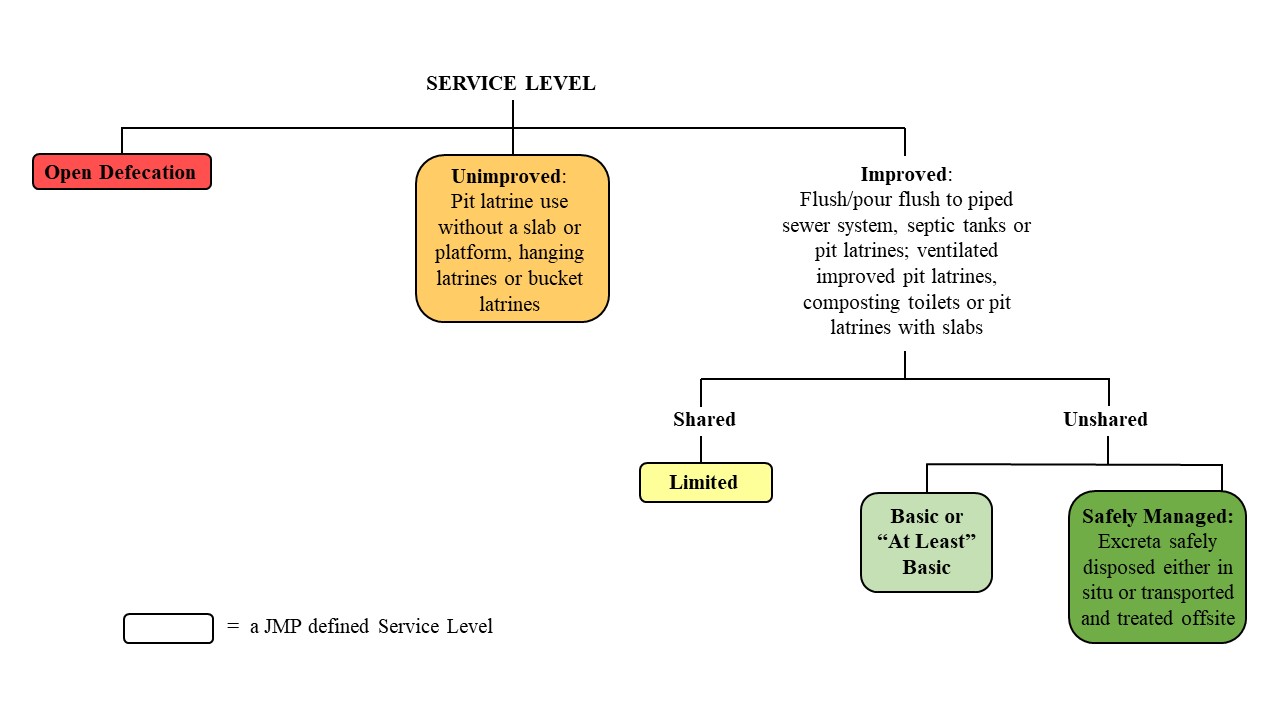


Figure 1. The JMP Service Level classifications and descriptions for onsite sanitation facilities.

**Sensitivity Analysis:**

**Number of Households using a Shared Facility**

To justify the decision to utilize three households as the standard to calculate the number of onsite sanitation facilities which are shared facilities, a sensitivity analysis was conducted. The total number of onsite sanitation facilities was calculated, using a range of households sharing a facility, from 1 household to 500 households. When compared with no shared facilities (1 household), two households had an 8% difference in the total number of onsite facilities, three households had an 11% difference, 10 households a 15% difference and 50-500 households had a 16% difference. Three households was selected as a midrange value, which was supported by suggestions from the literature.

Table 2. Sensitivity analysis for the number of households utilizing a shared sanitation facility. The percent difference represents the percent difference between unshared facilities (one household) up to 500 households using the same facility.

| **No. Households Sharing a Facility** | **Total No. Onsite Facilities** | **Percent Difference** |
| --- | --- | --- |
| 1 | 810,996,425 |  |
| 2 | 743,956,027 | 8% |
| 3 | 721,609,228 | 11% |
| 5 | 703,731,789 | 13% |
| 10 | 690,323,709 | 15% |
| 15 | 685,854,350 | 15% |
| 50 | 679,597,246 | 16% |
| 500 | 677,183,792 | 16% |
